# Strengthening the core health research capacity of national health systems helps build country resilience to epidemics: a cross-sectional survey

**DOI:** 10.12688/f1000research.24192.2

**Published:** 2020-06-29

**Authors:** Rony Zachariah, Dermot Maher, Abraham Aseffa, Mahnaz Vahedi, Pascal Launois, Mohammed Khogali, Garry Aslanyan, John C. Reeder

**Affiliations:** 1 UNICEF/UNDP/WORLD BANK/WHO Special Programme for Research and Training in Tropical Diseases, World Health Organisation (TDR), Geneva, 1211 Geneva 27, Switzerland

**Keywords:** COVID-19, Pandemic, Health systems, Training, Emergency preparedness

## Abstract

**Background: ** TDR, The Special Programme for Research and Training hosted at the World Health Organization, has long supported Low- and Middle-Income Countries in strengthening research capacity through three training programmes: the Postgraduate Training Scheme (PGTS), the Clinical Research and Development Fellowship (CRDF), and the Structured Operational Research Training InitiaTive (SORT IT). In the advent of the COVID-19 pandemic, we assessed whether those trained through these programmes were involved in the COVID-19 response and if so, in which area(s) of the emergency response they were applying their skills.

**Methods: **From the records for each training programme, we identified the individuals who had completed training during the relevant timespan of each programme: 1999-2018 for the CRDF scheme, 2015-2020 for PGTS, and 2009-2019 for SORT-IT. Between March and April 2020, we sent trainees an online questionnaire by e-mail.

**Results: **Out of 1254 trained, 1143 could be contacted and 699 responded to the survey. Of the latter, 411 were involved with the COVID-19 response, of whom 315 (77%) were applying their acquired skills in 85 countries. With some overlap between programmes, 84% of those trained through CRDF were applying their skills in 27 countries, 91% of those trained through PGTS were applying their skills in 19 countries, and through SORT IT, this was 73% in 62 countries.  Skills were being applied in various areas of the emergency response, including: emergency preparedness, situation analysis/surveillance, infection control and clinical management, data generation, mitigating the effect of COVID on the health system, and research.  Depending on the type of training programme, 26-74% were involved in implementation, operational or clinical research.

**Conclusion:** Research training programmes build research capacity and equip health workers with transferable core competencies and skillsets prior to epidemics. This becomes invaluable in building health system resilience at a time of pandemics.

## Introduction

One of the lessons that should have been learned from the 2014–2015 Ebola epidemic in West Africa, the largest and longest Ebola outbreak in history, was “the need to be better prepared for the next epidemic” ^1^. “The next epidemic” is happening now in 2020 and is the COVID-19 pandemic. This pandemic, of unprecedented global scale and impact, has tested the preparedness and resilience of every country. Among the many factors that contribute to preparedness and resilience, the capacity to undertake health research is a vital component of the response to infectious disease outbreaks. As we have seen from the current pandemic, all countries are at risk of infectious disease outbreaks and need to strengthen their capacity for a timely and effective research response. Capacity to undertake research varies widely among countries, reflecting the extent of investment and efforts to build and retain that capacity, usually over a long period of time. TDR, The Special Programme for Research and Training hosted at the World Health Organization (WHO), has long supported Low- and Middle-Income Countries (LMICs) in strengthening research capacity, through the range of activities needed to develop the necessary institutional base, research infrastructure, training programmes, career development pathways, research portfolio, regulatory frameworks and networks.

Fortunately, most countries, most of the time, do not have such outbreaks, so the opportunities for developing capacity for research through “on the job” learning during an outbreak are limited. This has two key implications. Firstly, developing capacity for research on infectious diseases, including: outbreaks takes place to a large extent before an outbreak (or between outbreaks) and is blind to the next specific infectious agent. Secondly, developing adaptable capacity for research on other health problems contributes to generic research capacity, which becomes applicable during infectious disease outbreaks. TDR supports a number of long-term programmes to strengthen capacity for research on infectious diseases: the Postgraduate Training Scheme (PGTS) on implementation research, the Clinical Research and Development Fellowship (CRDF) scheme on clinical research, and the Structured Operational Research Training InitiaTive (SORT IT) on operational research, a partnership-based initiative led by TDR and implemented in collaboration with various partners ( Box 1).

Box 1. **Brief description of three TDR-supported programmes to strengthen research capacity ^2^**

**Postgraduate Training Scheme:** TDR strengthens individual and institutional capacity for implementation research by supporting a network of seven universities in disease-endemic regions to train Master’s students on courses relevant to implementation research.

**Clinical Research and Development Fellowship (CRDF) scheme:** TDR supports placements of researchers with relevant partners (including pharmaceutical companies, product development partnerships, and public research institutions involved in clinical research) to strengthen their skills in clinical trials. The scheme started in 1999 with a small number of fellows and was scaled up in 2008 and 2014, with the support of the Bill & Melinda Gates Foundation (Gates Foundation) and in partnership with the International Federation of Pharmaceutical Manufacturers & Associations (IFPMA).

**Structured Operational Research Training Programme (SORT IT):** TDR coordinates a global partnership-based initiative to support countries and institutions to build sustainable operational research capacity. The target audience is front-line health workers from disease control programmes. The focus of training is on teaching practical skills for the generation of high quality, timely and disaggregated data for evidence-informed decision-making to improve public health.

Stimulated by examples of people who trained on these programmes and used the skills they gained to contribute to the COVID-19 response, we were interested to assess this more systematically. We therefore assessed whether those trained were involved in the COVID-19 response and if so, in which area(s) of the emergency response they were applying their skills.

## Methods

This was a cross-sectional survey that used three online questionnaires in English (one per programme, see *Extended* data ^3^; pre-tested on four selected trainees, following which minor changes were made to improve clarity) to gather information from the individuals who had been trained through the three programmes.

From the records of each training programme, we identified individuals who had completed training during the relevant timespan of each programme: 1999–2018 for the CRDF scheme, 2015–2020 for the PGTS, and 2009–2019 for SORT-IT. For those people with available contact details, we sent online questionnaires by e-mail (in March 2020 for SORT-IT and in April 2020 for the CRDF and PGTS) asking if they were currently involved in the COVID-19 response. We asked about the nature of their involvement, and if they were applying their acquired skills in responding to various key areas for tackling the pandemic.

The survey data was exported to Microsoft Excel for data analysis.

As part of monitoring and evaluation of TDR supported training programmes, routine online surveys are conducted to gather information for improving the quality and performance of such trainings. Participation in this survey was voluntary and individual consent was obtained for use of anonymized data for reporting and dissemination, including through publications, via the use of a yes/no tick box question within the questionnaires. As this study was part of routine monitoring and evaluation of a training programme, and potential ethical concerns were addressed (responders were all adults, response was voluntary, data were anonymized, personal identifiers were removed and no sensitive personal questions were included that could risk psychological or social harm), this was thus considered a minimal risk study and specific ethical approval for sending questionnaires was not required.

## Results

A total of 1143 individuals out of 1254 trained could be contacted; 699 responded to the survey. Table 1 shows the number of participants who reported involvement in the COVID-19 response, the number applying their acquired skills and the number of countries involved. Of 699 individuals who responded to the survey, 411 (59%; 152 female) reported involvement in the COVID-19 response, with 315 (77%) of the latter applying their acquired skills in 85 countries around the globe. With some overlap between programmes, 84% of those trained through CRDF were applying their skills in 27 countries, 91% of those trained through PGTS were applying their skills in 47 countries, and through SORT IT, this was 73% in 62 countries.

**Table 1.** Numbers involved with the COVID-19 response and applying skills gained through TDR supported training programmes.

Training programme	Trained n	Contacted n	Responded n	Involved in COVID-19 ^1^ n (%)	Applying skills ^2^ n (%)	Countries
Clinical Research and Development Fellowship scheme	111	104	68 (65)	45 (66)	38 (84)	27
Postgraduate Training Scheme	248	208	143 (69)	64 (45)	58 (91)	47
Structured Operational Research and Training Initiative	895	831	488 (59)	302 (62)	219 (73)	62
** TOTAL**	**1254**	**1143**	**699**	**411**	**315**	**85 ^3^**

^1^ Percentage is calculated using the number who responded as the denominator ^2^ Percentage is calculated using the number involved with COVID-19 as the denominator ^3^ This figure represents numbers of individual countries without overlaps between programmes. TDR: UNICEF, UNDP, World Bank, WHO Special Programme for Research and Training in Tropical Diseases.

Table 2 shows that trainees are applying their skills in a range of critical areas of the COVID-19 pandemic response. In terms of research, 74% of those trained through the CRDF scheme, were involved in clinical research, most commonly as a clinical trial manager. For PGTS, 45% were involved in implementation, operational research or clinical research, while 26% of trainees from the SORT IT programme were involved in implementation and/or clinical research.

**Table 2.** Areas of the COVID-19 response where trainees are applying skills from TDR supported training programmes.

Area of the COVID-19 response	Trainees applying skills ^1^		
	CRDF (N=38) n (%)	PGTS (N=58) n (%)	SORT IT (N=219) n (%)
Research	28 (74)	26 (45)	56 (26)
Critical preparedness and response	17 (45)	30 (52)	88 (40)
Situation analysis/surveillance	14 (37)	47 (81)	142 (65)
Infection control and clinical management	14 (37)	30 (52)	82 (37)
Data generation, analysis and reporting	17 (45)	39 (67)	119 (54)
Mitigating effect of COVID on other diseases	4 (10)	19 (33)	50 (23)
Other ^2^	6 (16)	39 (67)	15 (7)

^1^ Participants may be involved in more than one area (so percentages do not add up to 100%). CRDF: Clinical Research and Development Fellowship; PGTS: Post Graduate Training Scheme; SORT IT: Structured Operational Research and Training InitiaTive ^2^ E.g. Coordination, training, awareness raising etc.

## Discussion

The survey findings show that substantial numbers of health workers who were trained to improve their research capacity prior to the COVID-19 pandemic are currently involved in a wide range of emergency response activities.

This suggests that the respondents have used the specific skills they gained through trainings in combination with their abilities and knowledge as transferable competencies in responding to COVID-19 through a range of research and health system areas. This reinforces the value of TDR’s emphasis on developing core competencies (i.e. sets of skills combined with abilities and knowledge) through research training ^4, 5^. It also underscores the longer-term gains of investing in research capacity building programmes.

Regarding contribution to the research response to COVID-19, the high involvement of those trained through the CRDF scheme in clinical research (74%) is a practical example of applying the recommendation in the 2018 World Bank report “Money and microbes: strengthening clinical research capacity to prevent epidemics” concerning leveraging capacity-building from the private sector ^6^.

The significant involvement of trainees from PGTS and SORT IT in implementation and operational or clinical research shows that strengthening of core national research capacity before (or between) epidemics can make an important contribution to the timely mobilization of research resources during an epidemic.

The role played by TDR in supporting LMICs to strengthen capacity for clinical and implementation/operational research is in line with the WHO R&D blueprint ^1^ (the global strategy and preparedness plan that allows the rapid activation of research and development activities during epidemics). The development of the R&D blueprint in the aftermath of the 2014–2015 Ebola epidemic in West Africa was a recognition of the need to galvanize research, with the aim “to fast-track the availability of effective tests, vaccines and medicines that can be used to save lives and avert large scale crisis” ^1^. The focus on R&D needs to be complemented by efforts to promote implementation research, which helps to make sure that as new diagnostics, drugs and vaccines emerge from R&D pipelines they are evaluated in clinical trials and approved, they are made available to all who could benefit from them. Resources are needed to strengthen capacity for implementation/operational research, as well as for clinical research, in the LMICs where outbreaks are likely to occur ^7^.

Regarding contribution to the broad health system response to COVID-19, the survey results show that more than seven-in-ten of all trained prior to the COVID-19 pandemic are currently involved in a range of health system areas. These areas include: critical preparedness and response, situation analysis/surveillance, infection control and clinical management, data generation, analysis and reporting, and mitigating the effect of COVID-19 on other diseases. The research training has thus had wider benefits going beyond research, to provide generic skills that can be applied to a range of areas needed to tackle the pandemic. Limitations of this study are that we had no comparison group and we are unable to know the influence of non-response and social desirability bias. The extent and quality of contribution to the COVID response could also not be clearly defined.

In conclusion, the three TDR-supported training programmes have strengthened the health research capacity of health workers, thereby contributing not only to research but also to emergency preparedness and the broad health systems response to COVID-19. Such training programmes help build country resilience to epidemics.

## Data availability

### Underlying data

Open Science Framework: TDR_Training_COVID_Survey, https://doi.org/10.17605/OSF.IO/7YSZ2 ^3^.

This project contains the following underlying data:

- Dataset_1_CRDF_Survey_data_F1000.csv (Contains survey data on Clinical Research and development fellowships).
- Dataset_2. PGTS_Survey_data_F1000.csv (Contains survey data on Post Graduate Training Scheme)
- Dataset_3. SORT_IT_Survey_Data_F1000.csv (Contains survey data on Structured Operational Research and Training Initiative)

### Extended data

Open Science Framework: TDR_Training_COVID_Survey, https://doi.org/10.17605/OSF.IO/7YSZ2 ^3^.

The project contains the following extended data:

- Extended_data_questionnaire_1_CRDF.docx (Survey questionnaire for Clinical Research and development fellowships)
- Extended_data_questionnaire_2_PGTS.docx (Survey questionnaire for Post Graduate Training Scheme)
- Extended_data_questionnaire_3_SORT_IT.docx (Survey questionnaire for Structured Operational Research and Training Initiative)

Data are available under the terms of the Creative Commons Zero "No rights reserved" data waiver (CC0 1.0 Public domain dedication).
