# Supplementary material for: Strengthening the core health research capacity of national health systems helps build country resilience to epidemics: a cross-sectional survey
Source: F1000Res. 2020 Jun 29;9:583. Originally published 2020 Jun 9. [Version 2] doi: 10.12688/f1000research.24192.2 (PMC7308898; doi:10.12688/f1000research.24192.2)
Supplement: Peer review history

# Peer review: Reviewer response for version 2

**DOI:** 10.5256/f1000research.27669.r64561

**Published:** 2020-07-01

**Authors:** Yohanna Kambai Avong

**Affiliations:** 1 Institute of Human Virology, Abuja, Nigeria; 2 University of Technology Sydney, Sydney, NSW, Australia

The paper profoundly presents what the World Health Organization has been stressing on the need to develop research capacity at the supply side of healthcare. In the developing countries, training of researchers through traditional institutions, such as the University is one of the reasons we have very few qualified researchers since most people cannot afford the cost of attaining the universities.

 The paper is well written, concise, clear, and compelling. I strongly recommend the acceptance and immediate publication of the manuscript, to motivate the local onsite training of researchers and preparation for the next epidemic.

Is the work clearly and accurately presented and does it cite the current literature?

Yes

If applicable, is the statistical analysis and its interpretation appropriate?

Yes

Are all the source data underlying the results available to ensure full reproducibility?

Yes

Is the study design appropriate and is the work technically sound?

Yes

Are the conclusions drawn adequately supported by the results?

Yes

Are sufficient details of methods and analysis provided to allow replication by others?

Yes

Reviewer Expertise:

I am a public health pharmacist. My research interest is in Pharmacoepidemiologic research, with particular emphasis in drug safety and pharmacovigilance.

I confirm that I have read this submission and believe that I have an appropriate level of expertise to confirm that it is of an acceptable scientific standard.

# Peer review: Reviewer response for version 2

**DOI:** 10.5256/f1000research.27669.r65685

**Published:** 2020-06-30

**Authors:** Stephen Graham

**Affiliations:** 1 Burnet Institute, Melbourne, Vic, Australia

Is the work clearly and accurately presented and does it cite the current literature?

Yes

If applicable, is the statistical analysis and its interpretation appropriate?

Partly

Are all the source data underlying the results available to ensure full reproducibility?

Yes

Is the study design appropriate and is the work technically sound?

Yes

Are the conclusions drawn adequately supported by the results?

Partly

Are sufficient details of methods and analysis provided to allow replication by others?

Yes

Reviewer Expertise:

NA

I confirm that I have read this submission and believe that I have an appropriate level of expertise to confirm that it is of an acceptable scientific standard.

# Peer review: Reviewer response for version 1

**DOI:** 10.5256/f1000research.26686.r64566

**Published:** 2020-06-23

**Authors:** Desalegn Woldeyohannes

**Affiliations:** 1 Aklilu Lemma Institute of Pathobiology, Addis Ababa University, Addis Ababa, Ethiopia

The survey presents evidence of the usefulness of building up health research undertaking capacity to help contribute to strengthening the national health system resilience to epidemics. It addresses an important topic, given ongoing COVID-19 pandemic. The authors found that the research training programme, which was originally designed to strengthen the research capacity of health workers with the necessary competencies further contributing to health system resilience during COVID-19. It is great to see that researchers such as Rony and colleagues have evaluated the contribution of related training for strengthening the existing health system before or during epidemics. Such study findings will undoubtedly add new knowledge in the area, and could further be applied to reinforce health policy and practice.  

 The survey was well-designed, respective data analysis were performed largely with care. The presentation is excellent, with careful writing including tables along with the article help the follow-up to the reader. Below are more specific comments and suggestions by section:

**Abstract:**

 A very informative abstract, although data collection and instrument tool used to collect the data could also be included in the method section as well as in the text.

**Introduction:**

 It was a nice overview and set the scene beautifully for the survey. Be there any similar initiatives in the past that could add to the statement of the problem? In this aspect, I would suggest the inclusion of a few statements from research findings on the 2014-2015 Ebola outbreak with respect to how it impacted the health system operation in affected countries in West Africa. By doing so, the necessity of carrying out the current survey would better be justified.

**Methods:**

 The survey used a pre-tested questionnaire to collect data, and necessary changes were made to the questionnaire accordingly. Further to this, I would like the following points to be considered:

- Study design, only mentioned in the title.
- A few statements on how items in each questionnaire were developed, for example, whether benchmarked/adopted/modified, or what ways had been followed otherwise in developing them.   
- Were validity and reliability tests performed on the questionnaire in addition to pre-test so that assuring quality?
- How data were summarized and analysed? Were there any missing data reported, given the low response rate (56%) obtained for the survey, how authors took control of these while analysing the data?

 

**Results:**

 Well summarized using tables and text. What does ‘’other’’ refer to in Table 2? Good to mention it as a footnote as, for example the percentage of trainees applying skills in PGTS group for the same was significant, in fact second (67%) next to situation analysis/surveillance (81%), please list them all.

**Discussion:**

 While the dearth of literature/information in the area has largely prevented authors of making a robust scientific argument about the survey findings, their implications have clearly be presented towards practical application, and in national health policy enforcement for a similar pandemic. Further, would authors to expect similar results (percentage) if high response rate was obtained vis-à-vis the low response rate reported on the survey (56%)? This warrants discussion, perhaps as a limitation of the survey.

**Conclusion**:

 The conclusion was drawn adequately supported by the results.

Is the work clearly and accurately presented and does it cite the current literature?

Yes

If applicable, is the statistical analysis and its interpretation appropriate?

Yes

Are all the source data underlying the results available to ensure full reproducibility?

Yes

Is the study design appropriate and is the work technically sound?

Yes

Are the conclusions drawn adequately supported by the results?

Yes

Are sufficient details of methods and analysis provided to allow replication by others?

Yes

Reviewer Expertise:

Public health, biomedical science

I confirm that I have read this submission and believe that I have an appropriate level of expertise to confirm that it is of an acceptable scientific standard.

# Response

**Published:** 2020-06-24

**Authors:** Rony Zachariah

**Affiliations:** UNICEF7WORLD BANK7UNDP7WHO SPECIAL PROGRAMME FOR RESEARCH AND TRAINING IN TROPICAL DISEASES, Switzerland

**Point by point responses to peer reviewer comments**

**Reviewer 1:** **Dr.** **Desalegn Woldeyohannes**

Dear Dr Woldeyohannes,

Thank you very much for reviewing this paper and your useful suggestions. We have revised the manuscript and have provided a point-by-point response using bold font.

**Reviewer:** The survey presents evidence of the usefulness of building up health research undertaking capacity to help contribute to strengthening the national health system resilience to epidemics. It addresses an important topic, given ongoing COVID-19 pandemic. The authors found that the research training programme, which was originally designed to strengthen the research capacity of health workers with the necessary competencies further contributing to health system resilience during COVID-19. It is great to see that researchers such as Rony and colleagues have evaluated the contribution of related training for strengthening the existing health system before or during epidemics. Such study findings will undoubtedly add new knowledge in the area, and could further be applied to reinforce health policy and practice. 

The survey was well-designed, respective data analysis were performed largely with care. The presentation is excellent, with careful writing including tables along with the article help the follow-up to the reader. Below are more specific comments and suggestions by section:

**Response: Thank you for these encouraging comments.**

**Reviewer:** Abstract:

A very informative abstract, although data collection and instrument tool used to collect the data could also be included in the method section as well as in the text.

**Response: We have mentioned that we used an on-line questionnaire form.**

**Reviewer:** Introduction:

It was a nice overview and set the scene beautifully for the survey. Be there any similar initiatives in the past that could add to the statement of the problem? In this aspect, I would suggest the inclusion of a few statements from research findings on the 2014-2015 Ebola outbreak with respect to how it impacted the health system operation in affected countries in West Africa. By doing so, the necessity of carrying out the current survey would better be justified.

**Response: Unfortunately, there were no similar surveys done in relation to the 2014/2015 Ebola outbreak assessing the role of research training on building health system resilience in the affected countries.**

**Reviewer:** Methods:

The survey used a pre-tested questionnaire to collect data, and necessary changes were made to the questionnaire accordingly. Further to this, I would like the following points to be considered:

•Study design, only mentioned in the title.

**Response: We have added the study design to the Methods.**

**Reviewer:** •A few statements on how items in each questionnaire were developed, for example, whether benchmarked/adopted/modified, or what ways had been followed otherwise in developing them.  

**Response: We have added some brief details to the methods.**

**Reviewer:** •Were validity and reliability tests performed on the questionnaire in addition to pre-test so that assuring quality?

**Response: No, but we will consider this in the future. Thank you.**

**Reviewer: **•How data were summarized and analysed? Were there any missing data reported, given the low response rate (56%) obtained for the survey, how authors took control of these while analysing the data?

**Response. 699 (61%) out of 1143 responded to the survey and there might indeed be desirability bias. We have mentioned this in the limitations of the study. This limitation not with-standing, the overall numbers involved with the COVID-19 pandemic and the numbers using their skills is still encouraging.**

**Reviewer:** Results:

Well summarized using tables and text. What does ‘’other’’ refer to in Table 2? Good to mention it as a footnote as, for example the percentage of trainees applying skills in PGTS group for the same was significant, in fact second (67%) next to situation analysis/surveillance (81%), please list them all.

**Response: We have listed some of the items under ‘other’ in table 2**

**Reviewer:** Discussion:

While the dearth of literature/information in the area has largely prevented authors of making a robust scientific argument about the survey findings, their implications have clearly be presented towards practical application, and in national health policy enforcement for a similar pandemic. Further, would authors to expect similar results (percentage) if high response rate was obtained vis-à-vis the low response rate reported on the survey (56%)? This warrants discussion, perhaps as a limitation of the survey.

**Response. We have already alluded to this point in the limitations.**

**Reviewer:** Conclusion:

The conclusion was drawn adequately supported by the results.

**Response: Thank you.**

# Peer review: Reviewer response for version 1

**DOI:** 10.5256/f1000research.26686.r64563

**Published:** 2020-06-16

**Authors:** Stephen Graham

**Affiliations:** 1 Burnet Institute, Melbourne, Vic, Australia

Thanks for the opportunity to review this brief report which provides interesting and novel data, well written and presented.

 Some limitations of interpretation of the data could be highlighted in a short paragraph in the discussion, such as:

1. There is no group for comparison that represents the numbers (%) of those not trained who are involved in some way in the COVID response.
2. Could responders represent a biased sample, by nature of actively participating in this evaluation? The total numbers of past trainees involved may not be as high as “seven-in-ten”.
3. The extent and quality of contribution to the COVID response is not clearly defined

 

 Suggest in Introduction, the second sentence to delete “which” or “and”.

 Is this pandemic unprecedented in scale? Over 1.5 billion people infected (asymptomatic) with M.tb, around 10 million TB cases and 1.5 million TB-related deaths annually? What about the global death toll associated with the 1918 influenza pandemic?

Is the work clearly and accurately presented and does it cite the current literature?

Yes

If applicable, is the statistical analysis and its interpretation appropriate?

Partly

Are all the source data underlying the results available to ensure full reproducibility?

Yes

Is the study design appropriate and is the work technically sound?

Yes

Are the conclusions drawn adequately supported by the results?

Partly

Are sufficient details of methods and analysis provided to allow replication by others?

Yes

Reviewer Expertise:

NA

I confirm that I have read this submission and believe that I have an appropriate level of expertise to confirm that it is of an acceptable scientific standard.

# Response

**Published:** 2020-06-19

**Authors:** Rony Zachariah

**Affiliations:** UNICEF7WORLD BANK7UNDP7WHO SPECIAL PROGRAMME FOR RESEARCH AND TRAINING IN TROPICAL DISEASES, Switzerland

**Reviewer 2: Dr. Steve Graham**

 Dear Dr Steve Graham,

 Thank you very much indeed for having taken the time to review this paper and your useful comments and suggestions. We have revised the manuscript in line with your suggestions. We have provided a point-by-point response to your responses using bold font below

**Reviewer: **Thanks for the opportunity to review this brief report which provides interesting and novel data, well written and presented.

**Response: Thank you**

**Reviewer: **Some limitations of interpretation of the data could be highlighted in a short paragraph in the discussion, such as: There is no group for comparison that represents the numbers (%) of those not trained who are involved in some way in the COVID response. Could responders represent a biased sample, by nature of actively participating in this evaluation? The total numbers of past trainees involved may not be as high as “seven-in-ten”. The extent and quality of contribution to the COVID response is not clearly defined

**Response: We agree and have added a para on limitations and have covered thee above points**

**Reviewer:**  Suggest in Introduction, the second sentence to delete “which” or “and”.

**Response: done**

**Reviewer: **Is this pandemic unprecedented in scale? Over 1.5 billion people infected (asymptomatic) with M.tb, around 10 million TB cases and 1.5 million TB-related deaths annually? What about the global death toll associated with the 1918 influenza pandemic?

**Response: Since COVID-19 cases were first reported to WHO on 31 December 2019 over 8 million cases have now been reported and the numbers are climbing. We thus prefer to still regard this pandemic of a new human pathogen as unprecedented and we thank you for your kind understanding.**

# Peer review: Reviewer response for version 1

**DOI:** 10.5256/f1000research.26686.r64567

**Published:** 2020-06-15

**Authors:** Bethany Hedt-Gauthier

**Affiliations:** 1 Department of Global Health and Social Medicine, Harvard Medical School, Boston, MA, USA

Overall, this is a clear brief report with straight forward methods and justifiable conclusions. While it is possible that skills used were gained outside of the three training mechanisms, or that skills would have been gained elsewhere in the absence of these mechanisms, what is most important is that trainees identify that they are using skills in the COVID-19 response and the trainees attribute the skills to the specific training mechanisms described here.

 A few points to address:

- For the specific question, “what percent of trainees are currently using skills from training in COVID-19 response?” The more accurate denominator for this is all reporting trainees. To say that 77% of trainees are using skills for COVID response, when only 315 out of 699 reported as such, is not accurate. The authors clarify in the table with a footnote, but either they need to add a clause of: Of the 59% working on COVID-19 response, 77% report using their skills as part of that response. Or they need to recalculate and say that 45% report using their skills for the COVID-19 response. This is true for overall and by training type reports and should be reflected in the abstract and main text.
- Can the authors include reporting by gender and a short discussion? There has been expressed concern about how COVID response has been gendered, specifically with working women being excluded from participating in response either because of systemic issues or through home demands. I would be interested to see if this is an issue with trainees.
- Please add a statement about any influence of non-response and desirability bias.

Is the work clearly and accurately presented and does it cite the current literature?

Yes

If applicable, is the statistical analysis and its interpretation appropriate?

Yes

Are all the source data underlying the results available to ensure full reproducibility?

Yes

Is the study design appropriate and is the work technically sound?

Yes

Are the conclusions drawn adequately supported by the results?

Yes

Are sufficient details of methods and analysis provided to allow replication by others?

Yes

Reviewer Expertise:

Health systems research; research training and capacity building

I confirm that I have read this submission and believe that I have an appropriate level of expertise to confirm that it is of an acceptable scientific standard.

# Response

**Published:** 2020-06-19

**Authors:** Rony Zachariah

**Affiliations:** UNICEF7WORLD BANK7UNDP7WHO SPECIAL PROGRAMME FOR RESEARCH AND TRAINING IN TROPICAL DISEASES, Switzerland

**Point by point responses to peer reviewer comments**

**Reviewer 1: Dr.  Bethany Hedt**

 Dear Dr Bethany,

 Thank you very much for taking time to review this paper and your useful comments and suggestions. We have revised the manuscript in line with your suggestions. We have provided a point-by-point response to your comments and suggestions using bold font.

**Reviewer: **Overall, this is a clear brief report with straight forward methods and justifiable conclusions. While it is possible that skills used were gained outside of the three training mechanisms, or that skills would have been gained elsewhere in the absence of these mechanisms, what is most important is that trainees identify that they are using skills in the COVID-19 response and the trainees attribute the skills to the specific training mechanisms described here.

**Response: Thank you for this encouraging comment**

**Reviewer: **For the specific question, “what percent of trainees are currently using skills from training in COVID-19 response?” The more accurate denominator for this is all reporting trainees. To say that 77% of trainees are using skills for COVID response, when only 315 out of 699 reported as such, is not accurate.

**Response: We have now clearly indicated that for the 77% applying SORT IT skills, the denominator is “those involved with COVID-19” which is 411. Understandably, if one is not involved with COVID-19, it would be impossible to apply SORT IT skills. We thus prefer to keep the denominator of 411 and not 699.**

**Reviewer: **The authors clarify in the table with a footnote, but either they need to add a clause of: Of the 59% working on COVID-19 response, 77% report using their skills as part of that response. Or they need to recalculate and say that 45% report using their skills for the COVID-19 response. This is true for overall and by training type reports and should be reflected in the abstract and main text.

**Response. As suggested above, we have added the 59% (those involved with COVID-19) to the abstract and the results section.**

**Reviewer:** Can the authors include reporting by gender and a short discussion? There has been expressed concern about how COVID response has been gendered, specifically with working women being excluded from participating in response either because of systemic issues or through home demands. I would be interested to see if this is an issue with trainees.

**Response: Of the 411 involved with COVID, 152 (37%) were women. We have indicated this in the abstract and results section. As this proportion is more or less a reflection of the proportions trained by gender we have avoided further discussion.**

**Reviewer: ** Please add a statement about any influence of non-response and desirability bias.

**Response: In the Discussion, we have stated that “Limitations of this study are that we had no comparison group and we are unable to know the influence of non-response and social desirability bias.”**
